# After the *trans brain*: a critique of the neurobiological accounts of embodied trans* identities

**DOI:** 10.1007/s40656-023-00602-6

**Published:** 2024-02-02

**Authors:** Maite Arraiza Zabalegui

**Affiliations:** Department of Philosophy, Universidad del País Vasco/Euskal Herriko Unibertsitatea (UPV/EHU), Donostia/San Sebastián, Spain

**Keywords:** Trans* identities, Neurobiology, Trans brain, Mosaicism, Depathologisation

## Abstract

This paper critically analyses three main neurobiological hypotheses on trans* identities: the neurobiological theory about the origin of gender dysphoria, the neurodevelopmental cortical hypothesis, and the alternative hypothesis of self-referential thinking and body perception. In this study I focus then the attention on three elements: the issue of (de)pathologisation, the idea of the trans brain, and the aetiology of trans* identities. While the neurobiological theory about the origin of gender dysphoria and the neurodevelopmental cortical hypothesis claim the existence of the trans brain, each offering its own neurobiological depiction, the hypothesis of self-referential thinking and body perception doesn’t postulate a distinctive neurobiological trait for all trans* people. I problematize both portrayals of the trans brain departing from the findings and conceptualizations of the paradigm shifting brain mosaicism. Unlike the hypothesis of self-referential thinking and body perception that keeps the question of causation open, both the neurobiological theory about the origin of gender dysphoria and the neurodevelopmental cortical hypothesis situate the origin of trans* identities in the neurobiological domain. I challenge the biological deterministic framework in which this aetiology is inscribed from a dynamic processual entanglement perspective. Finally, concerning the issue of (de)pathologisation of trans* identities, an evolution can be seen in each of the hypothesis and among them, from the least to the most depathologising. However, I question their complete departure from a pathologising framework.

## Introduction

Transness has gained visibility in recent times (see Gossett et al., 2017). The work of the trans depathologisation movement and allies, Trans Studies scholars, and trans*^1^ activists in other fields has been instrumental in this regard. This incipient prominence has paralleled a growing, yet insufficient legal recognition of trans* people’s rights, and a proliferation of neuroscientific and neurobiological studies, attempting to explain and offer the keys of trans* identities.

Biological research, including most notably that focused on brain differences, has historically been used, and continues to be used, to legitimise, as well as to contest, social hierarchies and inequalities based on sex–gender, sexual orientation, and race. In the last three decades, particular emphasis has been placed on the search for brain differences between trans* and cis people. As Llaveria Caselles notes, “[t]he idea of the existence of neurological traits specific to trans people, is a culturally powerful narrative that has the potential to impact social perceptions, as well as legislative and medical regulations of trans people” (2021, para. 1). Given their multidimensional implications, a philosophical analysis of neurobiological accounts on trans* identities turns out to be a relevant and timely task in the current socio-political context.

In this paper, I take into account this context of the rise of essentialisms, in which trans* bodies have become a politic-epistemological battleground, where different conceptions on sex–gender identities are in contention, and argue that the idea of two brain types, the trans brain and the cis brain, is highly problematic. Moreover, I claim that the question regarding embodied trans* identities is a complex one, which cannot be reduced to neurobiological factors, nor to neurobiological causes. In doing so, I examine the main neurobiological theories on trans* identities to date, considering feminist and trans* approaches to neuroscientific, biological, philosophical, and political developments. To examine neuroscientific questions from a feminist standpoint expands and enriches them by adding a new stream of knowledge-making, increases rigour and accuracy, and becomes crucial when neuroscientists ask sex–gender questions (Bryant et al., 2019, para. 20).

The structure of the paper is as follows. In the first section, I introduce a neurobiological theory and a hypothesis about trans* identities, namely: the neurobiological theory about the origin of gender dysphoria and the neurodevelopmental cortical hypothesis. In the second section, I critically analyse these theories on the grounds of three major elements: the (de)pathologisation of trans* identities; the understanding of brains as sexually dimorphic (either the brain as a whole, i.e. the female brain and the male brain, or brain areas), giving way to the idea of the trans brain; and the biological deterministic framework where the aetiology and conceptualization of trans* identities are inscribed. In section four, I analyse the strengths and limitations of an alternative neurobiological hypothesis, that of self-referential thinking and body perception. In the last section, I present some concluding remarks.

## *The hunters of the trans brain:* theories on the neurobiological origins of trans* identities

Two theories that currently attempt to explain trans* identities are the neurobiological theory about the origin of gender dysphoria (NTOGD) and the neurodevelopmental cortical hypothesis (NCH). The NTOGD is embedded in the prolific organisational/activational (O/A) hypothesis, according to which testosterone, secreted by the foetus’ testes, masculinizes the foetus’ brain in utero, whilst its absence generates the female brain.^2^ According to this theory, brain structures and circuits that result from the interaction between sex hormones and neurons are the basis of sexually dimorphic behaviour (i.e. preferences, cognition or aggressiveness), sexual orientation, and gender identity. These aspects are permanently and irreversibly programmed, organised, and fixed in the perinatal brain. In puberty, hormone levels activate these hard-wired brain circuits and behavioural patterns.

From this framework, the NTOGD explains gender dysphoria as the result of an opposite sexual differentiation of the brain in the second half of pregnancy and the sexual differentiation of sexual organs during the first couple of months of pregnancy, which would create reversals in different sexually dimorphic brain structures (Savic et al., 2010, p. 57; Swaab, 2007, p. 441). This might happen due to immunological or genetic prenatal factors, such as chromosomal abnormalities and polymorphisms of the genes for androgen and oestrogens receptors, but the main cause are abnormal foetal hormone levels affecting the brain (Savic et al., 2010, pp. 48–49; Swaab & Bao, 2013, pp. 2979, 2985; Swaab et al., 2021, p. 430).

Dick Swaab’s team claims to have found this hormonally induced reversal in the volume and neuron number of two sexually dimorphic subcortical brain structures in postmortem studies. Kruijver et al. (2000) and Zhou et al. (1995) find it in the central subdivision of the bed nucleus of the stria terminalis (BSTc), and Garcia-Falgueras and Swaab (2008) in the third interstitial nucleus of the anterior hypothalamus (INAH3). Regarding the first interstitial nucleus of the anterior hypothalamus (INAH1), Garcia-Falgueras et al. (2011) find an intermediate neuron number compared to that of the male and female brain. These brain reversals lead them to affirm the existence of “the transsexual hypothalamus” (Garcia-Falgueras & Swaab, 2008, p. 3143), “the transsexual brain” (Kruijver et al., 2000, p. 2034; Swaab & Bao, 2013, p. 2976), and “the transgender brain” and “the cisgender brain” (Swaab et al., 2021, p. 435).

In addition to the fact that none of these findings has been replicated (Eliot et al., 2021, p. 669; Jordan-Young, 2010, p. 105)^3^, twelve brains of trans* people in all were enough to build one of the most widespread theories on transsexuality so far.

The neurodevelopmental cortical hypothesis (NCH) is both in line with and a refinement of the NTOGD, namely, of the central implication of hormones in brain sexual differentiation and the development of gender identity (Uribe et al., 2020, para. 2). On the basis that cortical thickness (Cth) develops during the first thirty years of life, the NCH upholds that there would be a slowdown (or a detention) in the cortical thinning process in ciswomen (CW), transwomen (TW) and transmen (TM), compared to that in cismen (CM), affecting different cortical regions and creating four distinct cortical phenotypes, one for each group: CW, CM, TW, and TM (Guillamon et al., 2016, p. 1637).^4^ The four phenotypes “might be due to differences in cortical development probably due to differences in the efficiency of sex hormone receptors” (Uribe et al., 2020, para. 2). In particular, the phenotypes of TW and TM may be causally linked to atypical effects of sex hormones or their metabolites in specific cortical regions (Guillamon et al., 2016, p. 1643) and to genes polymorphisms of androgen receptor and oestrogen receptors (Fernández et al., 2018, p. 165; Uribe et al., 2020, para. 2).

Considering that the cortical thinning process begins around puberty, in the case of both children who “have no problems with their gender identity” and those who persist in their gender dysphoria after puberty, gender identity doesn’t seem affected by sex hormone activation (Guillamon et al., 2016, p. 1637). Instead, with respect to the so-called “desisters”, hormone activational effects could play a significant role, either directly affecting the brain, cortical development, or guiding the development of secondary sex characteristics, which would “be perceived as congruent because of the brain changes that take place at this age” (Guillamon et al., 2016, p. 1637).

The NCH is formulated in a review paper without being empirically tested (Llaveria Caselles, 2021, para. 55). Its main ideas are nonetheless presented on the basis of four main MRI/DTI studies, three of them performed with transgender people not hormonally treated. In these studies, Rametti et al. (2011a, b) find that some of the fibres of the white matter that connect different cortical regions related to various skills, such as spatial and verbal ones, were masculinized and others defeminized in TM, while many of them were demasculinized in TW. Zubiaurre-Elorza et al. (2013) come across a thicker cerebral cortex in CW, TW, and TM than in CM, but it varied depending on the region.^5^ Thus, TW showed Cth feminization while TM showed subcortical masculinization in the putamen.^6^

Even if these studies acknowledge the complexity of the cerebral image of transgender people, “depending on the brain region studied and the kind of measures taken” (Zubiaurre-Elorza et al., 2013, p. 2860), the aforementioned skills, the fibres of the white matter, and different brain regions, such as the inferior parietal, the lingual gyrus or the putamen are defined as sexually dimorphic (Rametti et al., 2011a, p. 202; Zubiaurre-Elorza et al., 2013, pp. 2858–2859; Zubiaurre-Elorza et al., 2014, p. 1253).

## *Interlocking misconceptions*: a critical analysis of the NTOGD and the NCH

In this section, I offer a critical analysis of both the NTOGD and the NCH. In doing so, I will focus on three main elements: (1) the (de)pathologisation of trans* identities and how it is manifested in the theory and hypothesis examined; (2) the thesis of brain sexual dimorphism giving rise to the depiction of the trans brain; and (3) the biological deterministic framework where the aetiology of trans* identities is embedded.

Regarding the first element, pathologisation stigmatises trans* people, creating a breeding ground for discrimination and violence.^7^ Notwithstanding, it exceeds trans* identities, insofar as it is directly linked to the social conception of sex–gender.^8^ Thus, the understanding of sex–gender as fluid, multiple, and so, non-binary, which comprises a critique to the pressures imposed on bodies and subjectivities by cisheteropatriarchy is central for trans depathologisation movement, as well as for many trans* scholars and activists (see Halberstam, 2018; Missé and Coll Planas, 2010; Preciado, 2020; Spade, 2003; Suess Schwend, 2020; Transgender Europe, 2020). As Suess et al. explain,

the trans depathologisation framework introduces a paradigm shift in the conceptualization of gender identities: from conceiving gender transition as a mental disorder to recognizing it as a human right and expression of human diversity. From this perspective, the conflict is not situated in the individual trans person but in a society characterized by transphobia and gender binarism. Thus the contemporary concept of trans(s)exuality is analyzed as a culturally and historically specific construction. Furthermore, the ethnocentric and neocolonialist character of Western-biased psychiatric classifications is put into question for rendering invisible the cultural diversity of gender expressions and identities worldwide. (2014, pp. 74-75)

The discourse of depathologisation is gaining ground, as reflected in the formal depsycopathologisation of trans* identities in the *International Statistical Classification of Diseases and Related Health Problems Volume* 11 (ICD-11) (WHO, 2018). The ICD-11 removes trans-related categories from the chapter “Mental and Behavioural Disorders” and adds the code “Gender incongruence” in a new chapter called “Conditions related to sexual health” (Suess Schwend, 2020, para. 2). The trans depathologisation movement considers this change an improvement but demands the replacement of the category “gender incongruence” with a non-pathologising and stigmatising terminology –and its complete elimination in the case of children–, such as the descriptive “Health care related to gender transition” (GATE, 2019; Suess Schwend, 2020, paras. 29–30; Transgender Europe, 2020). Regarding the *Diagnostic and Statistical Manual of Mental Disorders V* (DSM-5) (APA, 2013), which changes the diagnostic category “Gender Identity Disorder” by “Gender Dysphoria”, trans depathologisation movement calls for the complete removal of trans-related categories, such as the latter or “Transvestic disorder” from DSM-5 (Davy et al., 2018, 20; Suess Schwend, 2020, paras. 27–28). In addition, depathologisation involves multiple social, legal, and medical changes, including gender self-determination and a shift in the trans health care model, from psychiatric assessment to an informed decision-making approach, of quality and public coverage (GATE, 2019; Suess Schwend, 2020, paras. 9, 33; Transgender Europe, 2020). As we will see, all these matters are relevant when analysing neurobiological theories about trans* identities.

The second element under scrutiny is brain sexual dimorphism, from which the idea of the trans brain in its different versions emerges. Both the NTOGD and the NCH are embedded in this framework, which has been recently contested by Daphna Joel and the transdisciplinary network of neurofeminist scholars NeuroGenderings, who embrace brain mosaicism. Brain mosaicism and its sheer amount of scientific evidence are part of “a new paradigm” (Fausto-Sterling, 2020, p. 338) that is deeply changing the way of understanding brain sex–gender differences.^9^ These researchers use the expression “sex–gender” to account for the intertwined and entangled interaction of sex and gender on the brain.^10^

Finally, the sex–gender thread carries two other inextricably linked constituents of the neurofeminist paradigm: entanglement and dynamism. These notions defy the biological deterministic framework in which the NTOGD and the NCH are embedded. Both concepts mean that “[e]ach individual’s behavioral and neural phenotype at the moment of experimentation is the dynamic product of a complex developmental process involving reciprocally influential interactions between genes, brain, social experience, and cultural context” (Rippon et al., 2014, p. 19). The idea of brain plasticity is central in this dynamism and has profound implications when it comes to understanding how entangled brains are with their environment (Rippon, 2019, xvi). In view of these clues and drawing from the dynamic process theory for the emergence, formation, and development of sex–gender identities (Fausto-Sterling, 2020, 2021), I will criticise the deterministic accounts of the NTOGD and the NCH that endorse a neurobiological origin of trans* identities. In doing so, I will reconceptualise both brains and identities from what I call “a dynamic processual entanglement perspective”.

### (De)pathologisation of trans* identities: still trapped in the old inheritance

In this section I will examine the narratives presented by the NTOGD and the NCH in their neurobiological accounts regarding trans* identities, the diagnostic categories employed, their understandings of sex–gender, and their clinical translatability.

The NTOGD conceptualises transsexuality as a failure or an error, as it can be seen in the expressions “being born in the wrong body/sex” (Swaab, 2007, p. 435; Zhou et al., 1995, p. 68) and “belonging to the opposite sex/gender” (Garcia-Falgueras et al., 2011, p. 3062; Garcia-Falgueras & Swaab, 2008, p. 3133; Swaab & Bao, 2013, p. 2983). Thus, the main aim is to ground this failure neurobiologically, which, as we have seen, it does by means of the opposite brain sexual differentiation and the idea of reversal. It is important to highlight that even if expressions such as those mentioned before have disappeared, the underlying assumption and the narrative remain the same.

In addition to the fact that the idea of reversal or inversion has been used since the end of the nineteenth century to describe sexualities and identities considered perverse, deviate or ill^11^, the theory includes other pathologically loaded words, such as “abnormal hormone levels”. It also employs diverse pathologising categories to refer to trans* identities that vary as well with the various diagnostic manuals. It is worth highlighting Swaab and Bao’s inclusion of transsexuality in their list of “neurological and psychiatric diseases”, together with multiple sclerosis, dementia, substance abuse or schizophrenia, among others (2013, p. 2981). Furthermore, it is significant that while in previous works the authors themselves call this theory “neurobiological theory about the origin of transsexuality” (Savic et al., 2010, p. 51; Swaab & Bao, 2013, p. 2987), they have recently renamed it as a “neurobiological theory about the origin of gender dysphoria” (Swaab et al., 2021, p. 427).^12^ This inscription in a pathologising framework is further reinforced by the claim of the need for a diagnostic biomarker regarding gender dysphoria, “since the diagnosis is at present only based on a description of the person’s own feelings” (Swaab et al., 2021, p. 438).^13^

To sum up, the search by Swaab’s team for a fundamental, essential brain difference in trans* people is embedded in a pathologising, dimorphic, and dichotomous framework, however much they assess “a great variability in all aspects of gender identity” (Swaab et al., 2021, p. 438).

Concerning the NCH, unlike the NTOGD it does not offer a pathologising narrative. In these works, trans* identities are referred to –although not always in an up-to-date manner–, in accordance with the successive versions of the diagnostic manuals. However, Uribe et al., (2020, para. 11) rename the diagnosis of the participants in the study, following the ICD-11 as “gender incongruence” to avoid stigmatisation of mental disorders. They also show a more open sex–gender conception that is not materialised in the research itself. In this manner, they classify identities in “transgender” and “cisgender” umbrellas. “Transgender” is “used to describe a diverse group of individuals whose gender identity is different (in varying degrees) from the sex assigned to them at birth” (Uribe et al., 2020, para. 1). But this diversity is reduced or subsumed by a binary and dichotomic research design, as well as by a binary, normative, and exclusionary diagnostic and health care access model. Indeed, all the people studied are characterised by a marked discrepancy between the sex assigned and the gender they identify with, namely, “the other” or “the opposite”, the desire for hormone therapy and surgery, and gender dysphoria (Uribe et al., 2020, paras. 1, 11, 60).

The result of this wide theoretical use of identity categories, yet restrictive use in practice is a bias in neurobiological research, which undermines the validity of the conclusions drawn. In this vein, in the international mega-analytic study carried out by ENIGMA Transgender Persons Working Group with the engagement of Guillamon and his collaborators, Mueller et al., (2021, p. 1127) acknowledge the need to include non-binary people and thus the limitations of their conclusions.^14^

Finally, regarding clinical translatability, the ultimate goal of this research of the neurobiology of transgender people is its clinical translation, namely, to “provide a normative framework that may become useful in clinical studies” (Mueller et al., 2021, p. 1123).

### *No turning back*: What is left of the *trans brain* without brain sexual dimorphism?

After having examined how the perspective of (de)pathologisation of trans* identities is entrenched in the literature that conforms both the NTOGD and the NCH, in this section I will outline the manner in which the concept of the trans brain is linked to brain sexual dimorphism. Departing from brain mosaicism, I will problematise the different depictions of the trans brain.

The NTOGD conceptualizes the BSTc, INAH3, and INAH1 –the latter only between ages 5 and 45– as sexually dimorphic, applying this dimorphism also to the whole brain: “the sexual dimorphic brain” (Kruijver et al., 2000, p. 2041) and “female brains/male brains” (Swaab & Bao, 2013, pp. 2979, 2985), the “male brain/feminine brain” (Swaab et al., 2021, p. 427). In contrast to these “cisgender brains”, the “transgender brain” (Swaab et al., 2021, p. 435) arises when reversals occur in the hypothalamic and adjacent structures mentioned above. In the case of the NCH, various brain regions including the inferior parietal, the lingual gyrus, and the putamen are defined as sexually dimorphic. The issue is to find whether the neuroanatomy of transgender persons is more similar to that of their biological sex, that of their gender identity (Rametti et al., 2011a, p. 199; Zubiaurre Elorza et al., 2013, p. 2855), “a combination of both, or something entirely different” (Mueller et al., 2021, p. 1123); namely, “the role of gender identity in contributing to this dimorphism” (Mueller et al., 2021, p. 1123). The specific combination of brain similarities and differences with respect to the sexually dimorphic brain differentiation constitutes the distinct and unique brain phenotypes of TW and TM, two of the four brain phenotypes. In this way, the NCH creates a distinction between cis brains and trans brains. As Mueller et al. put it: “transgenderism comes with its own unique profile” (2021, p. 1123).

Joel and the NeuroGenderings Network embrace the mosaic hypothesis, after testing it on a huge number of brains, on the basis of two key findings. First, they find that brain structures and connections are not sexually dimorphic, because “there is overlap between the distributions of females and of males on all currently known measures of the human brain that show sex/gender differences” (Joel, 2021, p. 165), and this overlap is extensive in most brain regions and connections, which undermines any possibility of distinguishing between a female and a male form for specific brain features (Joel, 2021, p. 170; Joel et al., 2015, p. 15,471). Considering, as Eliot et al. evince in their review of brain studies of the last three decades “Dump the «dimorphism»”, that once brain size is covaried^15^, there is almost no difference in the volume of subcortical and cortical structures between cis women and men, so that sex–gender explains about 1% of the total variance (2021, pp. 688–689). We cannot talk about a female and a male form even in relation to the measures that show the largest sex–gender differences found to date and, therefore, with less or relatively little overlapping, as the volume and number of neurons in the INAH1 and INAH3 (Eliot et al., 2021, p. 669; Joel, 2021, p. 169). The same can be said about the putamen, with a much smaller difference in volume, the BSTc, the inferior parietal, the lingual gyrus or Cth (Eliot et al., 2021, pp. 672–674; Joel & Fausto-Sterling, 2016, para. 1).

This substantial overlap has also been observed in most cognitive, social, and personality variables, so that group differences between women and men are small; even in characteristics such as spatial rotation or physical aggression there is a non-trivial overlap (Hyde, 2005, p. 590; Joel et al., 2015, p. 15,471; Rippon et al., 2014, para. 6). Besides, different studies indicate that these differences in mental rotation and in other spatial abilities are not present in early infancy, are smaller in children, and disappear when the brains of trained women and men are studied (Eliot et al., 2021, pp. 685–686).

The second finding of Joel and collaborators is that most brains are not internally consistent, but show great variability, i.e. cis women present features more common in cis men; cis men show features more common in cis women; and both, cis women and men, present features that are common in both (Joel, 2021, p. 166; Joel et al., 2015, p. 15,468). This means that brains, in general, are not sexually dimorphic, namely, they do not belong to two distinct types: male brain/female brain. The infeasibility of categorising them in these two distinct classes is “regardless of the cause of observed sex/gender differences” (Joel et al., 2015, p. 15,468). Far from this binary framework, brains are better characterised in one highly heterogeneous population, since each brain is conformed by a particular, unique, and ever-changing mosaic of regional and functional sex–gender differences (Joel, 2011, 2021; Joel & Fausto-Sterling, 2016; Joel et al., 2015).

The distinction between sex–gender differences and sexual dimorphism is important to understand what brain mosaicism means. The point is not that individual and group sex–gender differences do not exist. They do, and there are several. The point is that these individual differences do not consistently add up to the point of creating two distinct types of brains.^16^ Relevantly, individual sex–gender differences are present in people with diverse sex–gender identities.

What are the implications of brain mosaicism for the idea of the trans brain? Concerning the NTOGD, refuting the sexual dimorphism of BSTc and INAH3 entails the impossibility of their reversal, which is the basis of the trans brain.^17^ In fact, in light of the mosaic hypothesis, Garcia-Falgueras and Swaab co-analyse their results on INAH3 and INAH1 together with Joel, finding that, just with three brain measures, nine of the ten transgender women studied presented a mosaic brain (Joel et al., 2020, p. 162). Due to the overlap between transgender and cisgender people in the BSTc, INAH1, and INAH3, namely, “[s]ince two individuals with different gender or sexual identity may have the same brain measure, it is impossible to use these brain measures for diagnosis (e.g., of gender dysphoria)” (Joel et al., 2020, p. 164). However, despite their own findings, Swaab et al. continue to assert the existence of the transgender brain and the distinction between the cisgender and the transgender brain, the reversal in the BSTc, as well as the need to come across a biological marker for gender dysphoria (2021, pp. 435, 438).

With regard to the NCH, it bases its four brain phenotypes on differences in Cth between cis men and women, and between cis men and trans women and men.^18^ However, the existence of group differences between men and women in certain cortical regions—and here Joel et al. also mention Cth (2018, para. 43)—doesn’t allow us to talk about a male phenotype and a female phenotype. Firstly, because there is always overlap. Secondly, because “group-level sex–gender differences in specific brain features do not «add-up» to create two types of brains”, that is, two distinct brain phenotypes “one typical of females and the other typical of males” (2018, para. 2). Therefore, if regional group differences in Cth do not enable us to speak of two distinct brain phenotypes, that of cis women and that of cis men, they neither warrant talking of four distinct brain phenotypes, those mentioned plus trans women and trans men.

Apparently echoing the findings of brain mosaicism, while paradoxically still embracing neuroanatomical dimorphism, Mueller et al., (2021, p. 1127) do not find significant differences in Cth between transgender and cisgender persons, which is in contrast, as they themselves state, with previous results. This finding not only contradicts previous results, but it also defies the NCH and the unique four brain phenotypes. Nevertheless, they maintain this typology, on the grounds of differences they discover between transgender and cisgender persons in (sub)cortical brain volumes and surface area. This typology is taken as “somewhat consistent with recent empirical data from CM and CW that documented a «mosaic» pattern of a «neurophenotype» along the maleness-femaleness spectrum” (Mueller et al., 2021, p. 1127). Such consistency is nonetheless problematic due to at least two issues. Brain mosaicism is clear on the fact that brains are better characterised in one highly heterogeneous population than in two, since each brain, not each group –CW and CM– presents its own unique brain mosaic. It also claims that mosaic brains cannot be aligned along a male–female continuum, since they reside in a multidimensional space that cannot be meaningfully reduced to such a continuum (Joel, 2021, p. 166). While female-male continuum is useful for providing an account of the distributions of women and men on a particular brain feature, it fails as a description when various features or the whole brain are considered (Joel, 2021, pp. 171–172).^19^

It should be added that not only have the studies that shape the NCH not examined non binary people, but that they only analyse the brains of a subgroup of trans* people, i.e. those who desire hormone therapy and surgery, which implies the inapplicability of their conclusions to the entire spectrum of trans* people. This fact directly affects the typology of the four brain phenotypes and further compromises the idea of a “neuroanatomy of transgender identity” (Mueller et al., 2021).

Finally, there is the question of the conception of sex–gender identities and their neurobiological translatability. Sex–gender identities—whose social utility is another debate—are not internally consistent classes. Differences do not add up until creating distinct, self-closed, and internally consistent identities, but the “same” differences and similarities appear in various sex–gender identities. Differences and identities intersect, exceed, and constitute each other, problematising the neurobiological translatability of sex–gender identities. Therefore, like identities, brains do not form internally consistent types or classes, because differences and similarities appear in the brains of people with diverse sex–gender identities.^20^

### *On brains and identities*: challenging biological determinism from a dynamic processual entanglement perspective

Once I have problematised both portrayals of the trans brain, as a reversal of hypothalamic and adjacent structures and as two of the four brain phenotypes, here I will examine the aetiology of trans* identities, and sex–gender identities in general, proposed by the NTOGD and the NCH. I will also eschew an understanding of both brains and identities from a dynamic processual entanglement perspective.

Both the NTOGD and the NCH situate the cause of trans* identities, and sex–gender identities in general, in the neurobiological domain. According to the NTOGD, as intrauterine testosterone driven sexual differentiation of the brain takes place later than genital sexual differentiation of the genitals, these two processes can be influenced independently, resulting in gender dysphoria.^21^ Thus immunological factors, genetic factors, such as chromosomal abnormalities and polymorphisms of the genes for sex hormone receptors, as well as abnormal hormone influence would affect brain sexual differentiation prenatally, creating reversals in hypothalamic and adjacent structures and generating gender dysphoria (Savic et al., 2010, pp. 48–49; Swaab & Bao, 2013, pp. 2979, 2985; Swaab et al., 2021).^22^ Even if the endocrine disrupting antiepileptic drugs phenobarbital or diphantoin, and the synthetic oestrogen diethyletilbestrol (DES) taken during pregnancy also play their part (Savic et al., 2010, p. 49; Swaab & Bao, 2013, pp. 2983–2985; Swaab et al., 2021, p. 433), their effects would operate only on the foetus’s brain. The NTOGD denies the role of social postnatal environment in the occurrence of transsexuality and in the development of gender identity (see Savic et al., 2010, p. 57; Swaab, 2007, p. 436; Swaab & Bao, 2013, p. 2997; Swaab et al., 2021, p. 438).

Concerning the brain, this theory acknowledges context dependant epigenetic changes, namely, “depending on different activities in the genome, the environment, drug exposure, or social experience”, which regulate neurogenesis, neuronal apoptosis, and synaptic plasticity (Swaab et al., 2021, p. 432). Brain is characterised as a complex self-organising system that makes each brain unique and is involved in the variation of gender identity. Nonetheless, despite this alleged acknowledgement of brain dynamism, and contrary to the idea “that brain development after birth also has an important influence on gender identity”, the NTOGD concludes that gender identity arises in the womb (Swaab et al., 2021, p. 434). Its strong adherence to the brain hardwiring paradigm gets crystallised in their wording “programmed into the hardware of our brains for the rest of our lives” (Swaab et al., 2021, p. 438).^23^

In compliance with the NCH, which “assumes a perinatal action by androgens (or their metabolites)” (Guillamon et al., 2016, p. 1637), differences in the efficiency of sex hormone receptors would create differences in cortical development and four brain phenotypes, one for each variant of gender (Uribe et al., 2020, para. 2). Gender incongruence or transgender identities might be causally linked to atypical effects of sex hormones or their metabolites (Guillamon et al., 2016, p. 1643), gene polymorphisms of sex hormone receptors (Fernández et al., 2018), and epigenetic changes in DNA methylation (Ramirez et al., 2021). Ramirez et al. have recently found that the differential methylation of essential genes implied in brain neurodevelopment is involved within the aetiology of gender incongruence (2021, para. 38).^24^ They reaffirm the hypothesis of hormones, hormone receptors, genetics, and now also epigenetics as the origin of gender incongruence (Ramirez et al., 2021, para. 39). The NCH anecdotally mentions the contribution of environmental factors such as parental influences (Guillamon et al., 2016, p. 1616), without bestowing them real explanatory power on the development of gender incongruence.

Regarding the brain, the NCH emphasises the development of Cth and the thinning process of the cortex beginning in puberty, proposing “different thinning processes in different cortical regions” for each variant of gender (Guillamon et al., 2016, p. 1637). Nevertheless, the NCH basically sticks to the O/A hypothesis (Guillamon et al., 2016, pp. 1616–1617) modifying it slightly: gender identity, including trans* identities, wouldn’t be affected by sex hormone activation, except for those whose gender dysphoria fades after puberty.^25^

However, when addressing the emergence and development of sex–gender identities, including embodied trans* identities, social, political, cultural, historic, discursive, and environmental factors have to be taken into account. These factors encompass binary and cisheteronormative gender imperatives, stereotypes, and roles, so that these influence biology, in the same way that biology affects behaviour and identity, namely, all of them are profoundly entangled. Moreover, this occurs in a dynamic process. We depart from the conceptualization of Fausto-Sterling of identity as

a process rather than a thing. A process theory posits that identity self-organizes rather than being built according to a genetic blueprint. Nor is identity a fixed trait. Once stabilized it remains a dynamic entity, held more or less constant by a continuous back and forth between supporting experience and embodied responses. The fact that external experience and social context sustains and shapes identity means that it is fundamentally intersubjective rather than individual and autonomous. (2021, para. 43)

In her unfolding of a process-dynamic theory of the emergence and development of embodied sex–gender identities that contrasts with the examined deterministic narratives, Fausto-Sterling “describes identity as a cultural phenomenon that becomes woven into the body” (2020, p. 272). This means that identity is not located somewhere specific, not even the brain, but involves brain-body-world; it is a collective and emergent property of multiple entangled events. The self-organising complex system character of sex–gender identity implies that “it emerges from the activities of other complex systems”, such as cells, organisms (physiology and individual behaviour), intersubjective interactions, culture, and history (Fausto-Sterling, 2020, p. 303). Thus, sex–gender identity emerges in a dynamic process entailing the growth and development of the nervous system^26^; the embodiment of sex–gender norms and expectations in it, their storage in memory and their attachment to emotional development, through caregiver-infant dyadic interactions, interactions with others, and colours, toys, or clothing; as well as the development of sensorimotor, cognitive, and language skills linked to the emergence, acquisition, and mimesis of sex–gender-related knowledge, skills, and activities (Fausto-Sterling, 2020, pp. 298–313; 2021).^27^

Fausto-Sterling hypothesises that non-normative or non-binary identities arise from a range of sex–gender embodiment shaped by “the range of individual infant, parent, and infant-parent dyad differences in motor (and probably other) behaviors” (2021, para. 39). In other words, the behaviours of non-binary children simply are among an array of possible sex–gender identities.

The notions of entanglement and dynamism also characterise brains and hormones. Although steroid hormones (besides testosterone, oestrogens, and progesterone, among others) affect the brain thorough life, they do entangled with genetic, physiological, epigenetic, and environmental factors, including gender, socioeconomic status or education (Fine, 2017, p. 89; Joel et al., 2020, pp. 156, 160). These variables, moreover, operate and interact in multiple ways, generating different effects on different brain structures and features; consequently, even features affected by the same steroid can vary considerably (Joel et al., 2020, pp. 156–157; Joel, 2021, p. 166). The causal role of steroid hormones, not only in relation to sex–gender identities, but also to many sorts of behaviours has been profoundly problematised (see Fine, 2017; Jordan-Young, 2010; Jordan-Young & Karkazis, 2019; van Anders et al., 2015). As Fine et al. put it, “assumptions that brain circuitry is largely fixed by a genetic blueprint, that there is a unidirectional, causal pathway from genes to behavior via hormones and brains… have been widely rejected following conceptual and empirical upheavals in the relevant scientific fields”, demonstrating that individual behaviour, the behaviour of others, and environment “influence brain and behaviour through a reciprocal modulation of endocrine system”, contributing to brain plasticity (2013, pp. 550–551).

Despite the influence of early hormones on neural development, neurons, their connections, brain mosaics, and hormones themselves change and develop entangled with the environment in a lifelong process (Fine et al., 2013, p. 550; Joel & Fausto-Sterling, 2016, para. 10; Jordan-Young & Karkazis, 2019, pp. 28–29). Brain plasticity means that experiences, attitudes, norms, and behaviours in a binary and cisheteropatriarchal environment also shape and reshape brains. Accordingly, it is compulsory to take into account a variety of psycho-cultural factors when looking at any sort of measuring of structure or function of the brain, which only provides a glimpse of that brain in that particular moment (Rippon, 2019, pp. 335–336).

So, not only the insights and words “hardwired” and “permanent organisation” inadequately explain how early hormones affect the brain and unsuitably conceptualise sex–gender identities (Fausto-Sterling, 2020, p. 340; Jordan-Young, 2010, p. 288), but their development goes on throughout life in a dynamic process in which the socio-cultural merges with the biological. This process involves more or less stability or fluidity depending on bodies and subjectivities. While in some cases sex–gender identity or gender expression and/or the category employed for naming them change, and there are even those who alternate, with very diverse frequencies, different sex–gender identities—in all the cases described, also long after puberty–28, in many occasions the identity category remains constant over the whole lifetime. Nevertheless, changes in brain and body anatomy, physiology, subjectivity, experiences, also including the very identity, continue to take place.

## The hypothesis of self-referential thinking and body perception (HSRTBP). *Strengths and limits*

In this section I will analyse an alternative neurobiological hypothesis regarding trans* identities, that of self-referential thinking and body perception (HSRTBP). In doing so, I will bring back the previously addressed issues of (de)pathologisation of trans* identities, the trans brain, and the aetiology of trans* identities.

The HSRTBP decouples trans* identities from brain sexual dimorphism^29^ in its neurobiological account, bringing along other significant changes as well. It departs from studies indicating that we have an encoded image of our body in our brain and a brain network that codifies the self, so that these two large-scale networks interact. According to the HSRTBP, “GI is characterized by a functional disconnection between systems in the brain that process the perception of self (“self-referential”) and those that mediate own body perception (Majid et al., 2020; Manzouri et al., 2017)”, which could explain the discomfort with their bodies experienced by people with gender incongruence (Moody et al., 2021, para. 4).

Differences in coordinated activation and connections have been found in brain regions implicated in these networks, including the pregenual anterior cingulate cortex (pACC) and the amygdala, which would suggest the emotional significance of body-self alignment in transgender individuals (Feusner et al., 2017; Majid et al., 2020, p. 2905; Manzouri et al., 2017).^30^ The anatomical correlate of this disconnection would be a greater Cth in some of the regions mentioned (Kilpatrick et al., 2019; Manzouri et al., 2017).

Following DSM-5 and ICD-11—critiques displayed in subsect. 3.1. apply here too–, “gender dysphoria” and “gender incongruence” are employed as synonyms and defined as “significant distress and/or impairment due to a feeling of incongruence between a person’s experienced gender and their birth-assigned sex” (Moody et al., 2021, para. 1). However, notably they clarify that gender dysphoria or gender incongruence doesn’t characterise all transgender people, but a subset of them (Majid et al., 2020, p. 2898; Moody et al., 2021, para. 1).^31^ Brain functional disconnection wouldn’t be a distinctive trait generalizable to trans* people, a necessary condition in order to assess the existence of the trans brain.

By claiming that hormone therapy and surgeries present varied and not always positive results, Moody et al. (2021) create and test a machine learning model based on clinical and functional brain connectivity MRI data to predict individuals’ post-therapy body congruence and, thus, hormone therapy efficacy.^32^ They also propose its clinical use to “support and inform medical professionals whether or not to treat individuals with cross-sex hormone therapy” (Moody et al., 2021, para. 32). On the one hand, as the authors themselves explain, this claim is situated in the emergent approach of personalised or precision medicine (Moody et al., 2021, para. 2). On the other, the question arises as to which extent this biomarker application in the clinical practice related to trans* people does not run the risk of reinforcing medical authority and the role of diagnosis, paternalising trans* people and reducing their autonomy and decision-making capacity.^33^

The issue of causation remains open for the HSRTBP. Kilpatrick et al., (2019, pp. 3276–3277) and Manzouri and Savic (2019, pp. 2096–2097) insist on the reasons to believe that their findings reflect underlying factors rather than the effects of gender dysphoria, pointing that the neuroanatomical differences found in transgender people could be due to changes or differences in cortical development, and thus linked to its cause.^34^ However, given brain plasticity—including Cth and neural connectivity—the studies don’t permit the conclusion of whether this neurobiological substrate is innate or acquired (Manzouri & Savic, 2019, p. 2096; Manzouri et al., 2017, p. 1008). For Moody et al. (2021) “sociological, cultural, interpersonal, and biological factors are likely contributory” to gender incongruence. Among these interpersonal factors is the view that own body perception is shaped by a reciprocal interaction between one’s perception of physical appearance, rooted in self-observation and others’ reactions, and one’s body image in the brain (Majid et al., 2020, p. 2898; Manzouri & Savic, 2019, p. 2085). The alluded brain plasticity and multidimensionality could constitute the basis of a dynamic processual entanglement framework. Yet, as they are not central elements for the HSRTBP, this hypothesis is not able to explain how functional disconnection emerges in the brain of some trans* people.

From a cultural and social neuroscientific perspective that contrasts with the just brain centred neuroscientific research of the HSRTBP, Reubs Walsh and Gillian Einstein (2020) might shed some light in this regard. They blend this cultural and social neuroscientific perspective with a feminist philosophical view, all of which allows them to account for the intertwining of cultural, social, interpersonal, and neurobiological domains. In their approach to transgender embodiment, Walsh and Einstein (2020, p. 57) depart from previous informal distinctions between social dysphoria (the distress associated with being misgendered, namely, produced at the incongruity between one’s gender and other people’s perceptions of it) and bodily dysphoria (the distress in relation to bodily features that conflict with one’s perception of how a congruent gender embodiment should be in their case). On this basis, they reconceptualise bodily gender dysphoria experienced by some trans* persons as a manifestation (at least in part) of the harm that a cissexist, binary, and genitalocentric society does to the neural representation of the embodied self in the cortex (2020, p. 65).^35^ Together with the ways in which sex–related biology influences the brain and may affect brain’s representation of the body-shape, this damage and distress produced by such societal sex–gender model that constrains identity possibilities and pressures trans* people into a more binary gender transition, could be embodied by neuroplastic mechanisms generating bodily gender dysphoria (2020, p. 62).^36^ Thereby, according to Walsh and Einstein, “social gender dysphoria… may be a sufficient (but not necessary) condition for bodily gender dysphoria to emerge” (2020, p. 62).^37^

## Conclusion

In this critical analysis, three main neurobiological theories and hypothesis on trans* identities have been dissected, namely: the neurobiological theory about the origin of gender dysphoria (NTOGD), the neurodevelopmental cortical hypothesis (NCH), and the hypothesis of self-referential thinking and body perception (HSRTBP). In doing so, I have focused on three elements: (de)pathologisation of trans* identities, the aetiology of identities, and the portrayal of the trans brain.

Concerning the issue of (de)pathologisation, an evolution can be observed in each of the hypotheses scrutinised, as well as among them, from the most pathologising to the least pathologising or more depathologising. In this way, the NTOGD presents a highly pathologizing narrative, absent in both the NCH and the HSRTBP, while the latter acknowledges that only a subset of trans* people suffers from gender incongruence or dysphoria. Nonetheless, all of them employ psychiatric diagnostic categories or categories with psycopathological connotations, none of them has studied non-binary people, and the three advocate the need for a biomarker for the diagnosis of gender dysphoria or its clinical applicability, which problematises their complete departure from a pathologising framework. This current search for neurobiological markers and the vindication of their clinical applicability can be understood, not only linked to the blossoming approach of precision medicine, but also in relation to a social landscape that is taking relevant steps towards depathologisation of trans* identities, which has direct consequences for the medical authority.

Regarding the origin of trans* identities, whilst the NTOGD and the NCH situate it in the neurobiological domain, embracing mostly the O/A hypothesis, the HSRTBP keeps open the question of causation, endorsing brain plasticity, although without incorporating it as a central element. Several feminist neuroscientific, philosophical, and biological analyses highlight the relevancy of multiple dimensions entangled in a life-long dynamic process when it comes to addressing brain configuration, as well as the emergence and development of sex–gender identities, including trans* identities. The profound implications of brain plasticity for this multidimensional entanglement entail that cisheteropatriarcal norms, expectations, behaviours, and experiences shape and reshape brains, as well as embodied identities. Defying the notion of an inborn identity claimed by the NTOGD and the unmodifiable character of the experienced sex–gender identity suggested by the NCH, sex–gender identities develop in a dynamic life-long process.

The controversial concept of the trans brain is the final element scrutinised. Contrasting with the NTOGD and the NCH, which hold the distinction between trans and cis brains, each offering its own depiction of the trans brain, the HSRTBP brings with it the infeasibility of assessing a distinct neurobiological trait for all trans* people. The findings and postulates of brain mosaicism that contest the binary categorization of brains into the male and the female classes, allow us to problematise another couple of cerebral types, trans brain/cis brain; including the four cortical phenotypes version of the HNC, which maintains both by combining them. Thus, this paper further elaborates brain mosaicism, extending it to trans identities.

The problematicity of the neurobiological translatability of sex–gender identities into brain *types* is pointed out both from a strictly neurobiological point of view and in terms of essentialism. With regard to the latter, as Eliot et al., (2021, 690) highlight, “[t]he term «dimorphism» has potent heuristic value, reinforcing the belief of two categorically distinct organs” that have evolved to produce two psychologically distinct types of people designed to carry out different social tasks. Similarly, the idea of the trans brain and the distinction between trans and cis brains reveals the persistent pathologising inheritances and resistances to assume that trans* people are not *fundamentally* distinct of cis people, despite how different we all may be. This neurobiological inscription and rationale of trans* identities show an attempt to cling to scientific authority in the face of social changes that have begun to blur deep and rigid social hierarchies and divisions. Paraphrasing Rippon, perhaps it is time to give up the search for this kind of brain differences (2019, p. 333).

## Footnotes

1 Following authors such as Halberstam (2018) or Hayward and Weinstein (2015) I use the asterisk to give account of the multiplicity that gathers and floods “trans” category. “Trans*” encompasses many different sex–gender identities, expressions and life trajectories, such as transsexual, transgender, trans, transvestite, genderqueer, and gender fluid, among others, intersected with women, men or non-binary.

2 The formation of foetal gonads “occurs under the influence of a cascade of genes, starting with the sex-determining gene on the Y chromosome (*SRY*)”; while that of the sexual organs is mainly based on the influence or absence of androgens (Savic et al., 2010, p. 42; Swaab & Bao, 2013, p. 2978).

3 As Rebecca Jordan-Young points out, the study of Zhou et al. is an elaboration of the study of Kruijver et al., rather than a new one, “because 26 of the 34 subjects in the main analysis were the same as those studied by Zhou et al. (1995)” (2010, p. 105).

4 This paper and the studies referred below use the terminology “FtM and MtF transsexuals” and “women and men”. Uribe et al. (2020) change this nomenclature by “cisgender and transgender persons” and “cismen”, “ciswomen”, “transmen” and “transwomen”, also when summarising the results of their previous studies. In spite of the possible problematicity of this synonymy, for the sake of understandability I employ the last wording chosen by the authors here.

5 Specifically, in CW, the cortex appeared thicker in the frontal and parietal regions. In TM, it appeared thicker in the temporal and parietal lobes; and in TW in the orbitofrontal, insular, and medial occipital regions.

6 In these studies, average comparisons between the four groups were performed in the identified brain areas, failing to study variability between these groups. For the same methodological problem regarding neuroscientific research comparing cis women and cis men, see, for instance, Bryant et al. (2019); Rippon (2019, pp. 60, 351).

7 About this bond and the high rates of discrimination, precarization, and violence that suffer trans* people, see, for instance, James et al. (2016), Robles et al. (2016), Suess Schwend (2020), Transgender Europe (2021).

8 The social conception of sex-gender that imposes sex-gender coherence and only accepts two sex-genders brought with it the pathologisation of bodies, identities, trajectories, and expressions that subvert this coherence and transcend this binary conception.

9 Even if the “brain sex” or notions of brain anatomical differences between men and women have been criticised for more than three centuries, the development of neurosciences has given these indictments a new impulse. On the other hand, brain mosaicism was already known in neuroscience (Fausto-Sterling, 2020, p. 339; Rippon et al., 2014, paras. 12).

10 Some of them use the expression “sex-gender” following previous notions of the relationship between sex and gender elaborated by feminist authors such as Judith Butler (1990; 1993), Anne Fausto-Sterling (2000) or Donna Haraway (1991). See, for instance, Dussauge and Kaiser (2012, p. 212) and Kaiser (2012, p. 131).

11 See, for instance, also in regards to trajectories, expressions, and identities referred today as trans*, Krafft-Ebing (1892) and Benjamin (1966).

12 It should also be noted that this renaming takes place in a chapter in which they include the category “gender incongruence” of the ICD-11 as another possibility.

13 Note that, as the authors themselves suggest, the potential usefulness of the BSTc as a biomarker is compromised by the postmortem nature of their studies: “Concerning gender dysphoria, there is the finding of a reversal in the BSTc volume and cell number of the sex difference in transgender people. This has, however, so far not resulted in a specific and sensitive biological marker for gender identity in the brain in early development in vivo” (Swaab et al., 2021, p. 438).

14 This study includes a group of trans* people not diagnosed via clinical criteria, though most of the participants were diagnosed using pathologising categories.

15 Since bodies of adult cis men are on average 10–11% bigger than those of cis women—affecting all organs, though differently—their brain is around 10–11% bigger (Eliot et al, 2021, p. 669).

16 Even the argument of sex prediction departing from brain data seems problematic in order to justify the categorization of the male and the female brain. See in this regard Joel (2021) and Sanchis-Segura et al. (2020).

17 It should be added to this that the function of INAH3 is unknown and it is not clear its relationship, if there is any, with gender identity (Eliot et al., 2021, p. 688; Jordan-Young, 2010, p. 50). Besides brain sex-gender differences not necessarily being translatable into different functions, most brain regions are multitaskers, brain functions are usually not located in a particular brain structure but distributed over circuits of many interacting brain areas, and the same behaviour could be generated by somewhat different neural networks in different people (Fine et al., 2019, para. 40; Joel & Fausto-Sterling, 2016, para. 12; Joel et al., 2020, p. 164).

18 Nonetheless, not all the cortical areas in which these differences were found (see footnote 5) seem to be related to identity, much less gender identity, which problematizes the underlying rationale of this research.

19 The number of different brain mosaics with the same score or number of female-ended, male-ended, and intermediate characteristics is huge. So, on the one hand, such an alignment implies that many brains are ill defined, since each score may contain many different brain mosaics. On the other, it denotes a misconception of brains, as two mosaics with the same score or number of features may be different from one another and more similar to other mosaics with different scores (Joel, 2021, p. 172). In this regard, both the concept of the female/male brain (apart from being inadequate, due to the lack of internal consistency of most brains) and assigning a brain score on a female–male continuum give very little information about that particular brain’s structure, functioning, and about that person and their behaviors, preferences, etc.

20 Even if this paper embraces a non binary conception of sex-gender identities, it doesn’t postulate that the binary identity conception is overcome by brain mosaicism. However, both the mosaic hypothesis and a non-binary sex-gender conception point to multiplicity or variability as a core issue. How this multiplicity is organized and classified when it comes to sex-gender identity categories is a social matter. What the paper postulates is that the concept of the mosaic brains can and should also be applied in the case of trans people, be they binary or not. Thus, the conception of brain *types* is problematized, both from a strictly neuroanatomical perspective and, as the paper concludes, in terms of essentialism.

21 Apart from hormones, genetics and epigenetics are also linked to the aetiology of gender identity. “The process by which early exposure to gonadal hormones organises the brain and creates the «cellular memory» elicits a sexual dimorphic response in adulthood. This process is also thought to occur through epigenetic modifications” (Swaab et al., 2021, p. 432).

22 For more details on the factors mentioned see the references cited.

23 Note that, in the co-authored paper together with Joel, Swaab previously stated the impossibility of determining if differences in brain structures are programmed and linked to the cause of gender identity, or the result of the experiences of people with different gender identity (Joel et al., 2020, p. 164).

24 Besides the small sample size, the study doesn’t take into account “other factors with a known influence on the DNA methylome”, such as sleep profile, active/sedentary lifestyle, nutritional habits, and early life adversity (Ramirez et al., 2021, p. 41).

25 Particularly clear in this sense is Guillamon’s following explanation: “[I]dentity is determined by a biological question… For most people, gender identity is formed in the first two or three years of life, but… there are people who reach puberty and are still not very clear about it… a significant proportion of boys who reach puberty claiming to be girls or vice versa, later give up. Later on, they become aware of being a homosexual person in accordance with their identity. Some reach puberty without having previously consolidated their identity, and sometimes identity is confused with sexual orientation” (Mediavilla, 2022, own translation). Nevertheless Guillamon is clear on the fact that “the gender identity that is experienced is unmodifiable” (2021, own translation).

26 This includes synaptogenesis and synaptic pruning, myelination, the development of different cortical areas, and connectome (Fausto-Sterling, 2021, para. 19). Although plasticity is a life-long brain feature, it is even more striking in early development (Rippon, 2019, pp. 149-150).

27 For the timings of these entangled events from birth—and before—to three years of life, when the majority of children assess a sex–gender identity, see the references cited. The sense of self stabilises via autopoiesis, operational closure giving rise to a global property, identity, “without requiring «a central controller» such as an identity gene or a special group of identity brain cells”; but it can’t be reached without engaging in dyadic interactions, specific sorts of sex–gendered activities, and the world’s meanings (Fausto-Sterling, 2021, para. 37). It should be kept in mind as well the historical character of the Western idea of gender identity itself, which can be rooted in the end of nineteenth century (Mak, 2012).

28 Indeed, the emergent property and self-organising system character of sex-gender identity accounts for the possibility of qualitative change occurring later on in life, and more than once.

29 In spite of this detachment, brain sexual dimorphism is claimed (see Kilpatrick et al., 2019, p. 3270; Manzouri et al., 2017, pp. 999, 1007; Manzouri & Savic, 2019).

30 Regions also encompass the medial prefrontal cortex, the occipital cortex, the precuneus, and the posterior cingulate cortex (Feusner et al., 2017; Majid et al., 2020; Manzouri et al., 2017).

31 Earlier studies of the hypothesis, though, use “transgender” and “gender dysphoria” as rather synonyms (see Kilpatrick et al., 2019; Manzouri & Savic, 2019).

32 Albeit the predictive model was solely based on neurobiological features, Moody et al. point out the potential of multivariate techniques to “provide additional insight into not only the neurobiological bases but also the sociological, cultural, and psychological bases of gender and body satisfaction” (2021, para. 25).

33 Indeed, this kind of studies have been denounced by many LGBTQ + organisations, among other things for being “suggestive of a search for medical «cure,» which can open the door for more gatekeeping and restrictive policies and practices in relation to access to gender-affirming care” (California LGBTQ Health and Human Services Network, 2021).

34 In this line, Uribe et al. (2020), who study regional and global connectivity within functional networks in transgender persons, conclude the consistency and complementarity of the NCH and the HSRTBP.

35 Living life as a trans* person in a cissexist, binary, and genitalocentric society “means your perception of yourself conflicts with the combination of society’s dominant narrative about gender, and the body you occupy” (Walsh & Einstein, 2020, p. 57). As Butler explains what is at stake here is cultural intelligibility and, thus, livability of social lives (1993). In this line, Rippon stresses the centrality of the drive to be social to human behaviour, comparing the hurt produced by being excluded with an electric shock (2019, p. 128).

36 Indeed, Rippon highlights the relevance of social pain and rejection as brain-changing elements (2019, pp. 125, 128). Together with the prefrontal cortex, particularly the medial prefrontal cortex, which is involved in self-identity, and the amygdala, related to emotional processing and expression, the anterior cingulate cortex (ACC) seems to be linked to social pain and rejection (Rippon, 2019, pp. 124–129).

37 The proposal of Walsh and Einstein is consistent with the findings of Robles et al. (2016) on distress and impairment. Their data analysis with multivariate logistic regression models indicates that distress and dysfunction are strongly predicted by experiences of social rejection and violence. Of the variables of gender incongruence, discomfort with bodily features, physical and behavioural changes undertaken, and asking to be referred to as one’s gender, only the latter was a significant predictor (Robles et al., 2016, p. 856). In their study published in *The Lancet Psychiatry* the authors conclude that “[d]istress and dysfunction… were more strongly predicted by experiences of social rejection and violence than by gender incongruence, consistent with the perspective that these reflect the result of stigmatisation and maltreatment rather than integral aspects of transgender identity” (Robles et al., 2016, p. 857).
